# A High-Strength Solid Oxide Fuel Cell Supported by an Ordered Porous Cathode Membrane

**DOI:** 10.3390/membranes14020044

**Published:** 2024-02-04

**Authors:** Ting Chen, Huilin Zhang, Guozhu Zheng, Qiang Xue, Zuzhi Huang, Yucun Zhou, Shaorong Wang

**Affiliations:** 1 School of Chemistry and Chemical Engineering, China University of Mining and Technology, 1 Daxue Street, Xuzhou 221116, China; chenting@cumt.edu.cn (T.C.); ts23040226p31@cumt.edu.cn (Q.X.); 2 Jiangxi Key Laboratory of Surface Engineering, School of Materials and Energy, Jiangxi Science and Technology Normal University, Nanchang 330013, China; 3 Beijing Huairou Laboratory, Beijing 101400, China

**Keywords:** solid oxide fuel cell, ordered cathode membrane, high strength, phase inversion, DRT analysis

## Abstract

The phase inversion tape casting has been widely used to fabricate open straight porous supports for solid oxide fuel cells (SOFCs), which can offer better gas transmission and minimize the concentration polarization. However, the overall weak strength of the macro-porous structure still limits the applications of these SOFCs. In this work, a novel SOFC supported by an ordered porous cathode membrane with a four-layer configuration containing a finger-like porous 3 mol% yttria- stabilized zirconia (3YSZ)-La_0.8_Sr_0.2_Co_0.6_Fe_0.4_O_3−δ_ (LSCF) catalyst, porous 8 mol% yttria-stabilized zirconia (8YSZ)-LSCF catalyst, and dense 8YSZ porous 8YSZ-NiO catalyst is successfully prepared by the phase inversion tape casting, dip-coating, co-sintering, and impregnation process. The flexural strength of the open straight porous 3YSZ membrane is as high as 131.95 MPa, which meets the requirement for SOFCs. The cathode-supported single cell shows a peak power density of 540 mW cm^−2^ at 850 °C using H_2_ as the fuel. The degradation mechanism of the SOFC is investigated by the combination of microstructure characterization and distribution of relaxation times (DRT) analysis.

## 1. Introduction

The development of sustainable, clean energy conversion and storage systems has drawn extensive attention all over the world. Among all of the electrochemical devices, solid oxide fuel cells (SOFCs) have garnered significant interest due to their exceptional energy conversion efficiency, environmental friendliness, and wide fuel adaptability [1,2,3]. Three different types of cells, i.e., anode-supported, electrolyte-supported, and cathode-supported SOFCs, have been widely reported for their unique structures and adaptability to operate in a variety of situations [4,5]. Anode-supported SOFCs are the most widely studied configuration because of their high electrochemical performance at intermediate temperatures and the relatively mature fabrication process [6]. However, the insufficient redox stability of the Ni-based anode, as well as the concerns related to carbon deposition and sulfur contaminants, have limited its application for the direct use of hydrocarbon fuels [7]. Electrolyte-supported SOFCs typically have high stability but poor electrochemical performance due to the thick electrolyte layer, which is necessary to maintain the mechanical strength [8]. Therefore, the cathode-supported SOFC is proposed due to its benefits of great fuel flexibility, a low sintering temperature, high durability, wider selection of anode materials, and potentially lower production costs compared to other designs [9,10]. Additionally, cathode-supported SOFCs may offer improved resistance to certain fuel contaminants, such as sulfur, making them suitable for a wider range of applications [11].

Nonetheless, the catalytic activity of the cathode electrode for cathode-supported SOFCs still needs to be improved. Because the thicker cathode support layer can lead to worse gas diffusion and larger concentration polarization resistance, sufficient porosity for the cathode support is strongly necessary for oxygen reduction reaction (ORR) sites [12,13]. In contrast to the irregularities and low permeability of the pores made with traditional pore formers (such as carbon, graphite, flour, and starch), the phase inversion method is an effective method to prepare a finger-like, open, straight-hole support with better gas diffusion ability [14,15]. The finger-like hole structure can effectively solve the problem of mass transfer resistance and offer an easier path for gas diffusion [16,17]. In our previous work, a (La_0.8_Sr_0.2_)_0.95_MnO_3−δ_ (LSM)-supported SOFC was fabricated by tape casting using wheat starch as the pore former, and the concentration polarization resistance was observed during the measurement [18]. Zhang reported a LSM-8YSZ-supported SOFC fabricated by the phase inversion tape casting method and showed a peak power density of 464 mW cm^−2^ at 850 °C [19]. Meng reported a 8YSZ/LSM-8YSZ dual-layer hollow-fiber-supported tubular SOFC and achieved a maximum power density of 290 mW cm^−2^ at 850 °C [20]. Although the gas diffusion property and electrochemical performance of cathode-membrane-supported cells have been significantly improved using the phase inversion method, at present, the mechanical strength of the straight-hole support prepared by the phase inversion method is undesirable. Shi adopted 3YSZ as the support and displayed great mechanical strength and electrochemical performance, but it was applied to anode-supported SOFCs [21]. In addition, it should be noted that the cathode materials for cathode-supported SOFCs are mostly perovskite structure composite oxides doped with alkaline earth metals (such as La_1−x_Sr_x_MnO_3_, LSM). In order to maintain the electrochemical activity of cathode materials for oxygen reduction reactions and avoid chemical reactions with electrolyte materials at high temperatures, the sintering temperature needs to be lower than 1300 °C, which may also reduce the mechanical strength of the fuel cell [15,16]. On the other hand, the interface bonding between the cathode and electrolyte layers is a crucial factor affecting the electrochemical performance and stability of SOFCs during practical applications. Delamination and degradation issues can arise due to the mismatched thermal expansion coefficients and chemical compatibility between the cathode, electrolyte, and other cell components [22,23].

In order to solve the above problems, a novel high-strength four-layer cathode-supported SOFC with a configuration of finger-like porous 3 mol% yttria-stabilized zirconia (3YSZ)-La_0.8_Sr_0.2_Co_0.6_Fe_0.4_O_3−δ_ (LSCF) catalyst, porous 8 mol% yttria-stabilized zirconia (8YSZ)-LSCF catalyst, and dense 8YSZ porous 8YSZ-NiO catalyst is proposed and prepared via the phase inversion tape casting, dip-coating, co-sintering, and impregnation method in this work. The open, straight-hole 3YSZ supporting membrane, which has not been used for a cathode support before, could offer excellent mechanical performance. The active cathode prepared by the impregnation method can be calcined at a lower temperature to obtain a nano-sized cathode with more triple phase boundary (TPB) active sites for performance enhancement [24,25]. The porous 8YSZ/dense 8YSZ/porous 8YSZ could be co-sintered in one step and offer excellent interface bonding since there is no thermomechanical stability issue between 8YSZ materials. The use of the impregnation method could also reduce the fabrication temperature, which can avoid the possible chemical reaction between the cathode and electrolyte over 1000 °C; therefore, a barrier layer of Gd-doped CeO_2_ (GDC) is not necessary [26]. Commercial LSCF and NiO are used as the cathode and anode catalyst, respectively, because of their excellent catalytic activity and their feasibility for widespread application. The electrochemical performance and stability of the single cells are evaluated. Additionally, the distribution of relaxation times (DRT) method is utilized to analyze the impedance spectra measured at different temperatures and operation times, providing a better understanding of the electrochemical reactions of the SOFCs.

## 2. Experiment

### 2.1. Cell Fabrication

An ordered porous cathode-supported single cell consisting of a 3YSZ-La_0.8_Sr_0.2_Co_0.6_Fe_0.4_O_3−δ_ (LSCF) support layer, 8YSZ-LSCF cathode functional layer, dense 8YSZ electrolyte layer, and 8YSZ-NiO anode functional layer was fabricated via the phase inversion tape casting, lamination, co-sintering, and impregnation method. The phase inversion method was used to fabricate the finger-like 3YSZ support membrane. Firstly, the organic solution was prepared by mixing the binder of polyether sulfone (PESf), the dispersant of polyvinyl pyrrolidone (PVP), and the solvent of 1-2-methylpyrrolianone (NMP) with a weight ratio of 4:1:20. Secondly, 3YSZ and polymethyl methacrylate (PMMA) were added into the ball milling tank with a weight ratio of 20:1 and subsequently added into the prepared organic solution mentioned above, and the weight ratio of powder to organic solvent was controlled at 1.5:1. Then, the slurry was obtained after adding appropriate ball milling beads and milling for 4 h. Thirdly, after the slurry was filtrated and vacuum pumped, the slurry was tape-cast through a doctor blade with a gap of 1 mm. The resulting tape was immediately submerged in water for the organic phase inversion process. The 3YSZ cathode support was eventually taken out from the water and dried. Finally, the support layer was obtained by cutting the tape into wafers and subjecting them to pre-sintering at 1100 °C for 4 h.

A symmetrical cell with three layers of porous 8YSZ, dense 8YSZ, and porous 8YSZ was purchased from commercial sources in China. The LSCF catalyst with 10 wt%, 15 wt%, and 20 wt% loading to the whole symmetrical cell was impregnated and calcined at 800 °C and 850 °C, respectively. For single cell fabrication, the porous 8YSZ layer, dense 8YSZ layer, and porous 8YSZ layer were fabricated by a dip-coating process. The preparation of the porous 8YSZ and dense 8YSZ slurries has been reported in our previous work [19]. Then, the four-layer structure of porous 3YSZ, porous 8YSZ, dense 8YSZ, and porous 8YSZ was co-sintered at 1400 °C for 5 h. The resulting cell skeleton was obtained after polishing the surface sponge layer of the 3YSZ support with exposed finger holes.

NiO as the anode catalyst and LSCF as the cathode catalyst were impregnated into the porous layers, respectively. Both the anode and cathode impregnation solutions were prepared by the citric acid complexation method, consistent with previous nitrate solution preparation [19]. The appropriate amounts of La(NO_3_)_3_·6H_2_O, Co(NO_3_)_2_·6H_2_O, Sr(NO_3_)_2_, Fe(NO_3_)_3_·9H2O, and citric acid were dissolved in a 30% ethanol–70% deionized water solution, with a controlled molar ratio of nitrate to metal ions of 1:1. Then, the LSCF nitrate precursor solution (1 mol/L) was injected into the porous 3YSZ-8YSZ side of the obtained skeleton and the Ni(NO_3_)_2_ precursor solution (1 mol/L) was added into the porous 8YSZ layer. Heat treatment was conducted at 450 °C for 1 h after each impregnation. The single cell was finally obtained after sintering at 800 °C for 2 h to decompose the nitrates. The final loadings of LSCF and NiO were approximately 15 wt% and 2 wt% of the weight of the whole cell, respectively.

### 2.2. Electrochemical Performance and Characterization

The strength of the cathode 3YSZ support with a size of 20 mm × 30 mm was tested by the three-point bending method. The morphology of the cross-section and bottom view of the 3YSZ support and the microstructure of single cells were characterized by an environmental scanning electron microscope (ESEM, FEI Quanta 250, Hillsboro, FL, USA). The electrochemical impedance spectroscopy (EIS) of symmetrical cells was performed at 650–850 °C both in air and O_2_ with a flux of 40 mL/min. Additionally, the current–voltage–power (I-V-P) curve and EIS of the single cell were obtained by an electrochemical workstation (SP-300, Bio-Logic, Seyssinet-Pariset, France) at 650–850 °C. The flow rate of both H_2_ and air was controlled at 40 mL/min. The EIS measurement was performed in a frequency range of 5 MHz to 50 mHz with a voltage amplitude of 10 mV. The effective anode area of the single cell was 0.28 cm^2^. Furthermore, the distribution of relaxation times (DRT) method was used to study the degradation mechanism of single cells. The AC impedance data of fuel cells were fitted by DRT peaks through the Matlab software (R2028a) [27].

## 3. Results and Discussion

### 3.1. Flexural Strength of the 3YSZ Support Membrane

**Figure 1:**
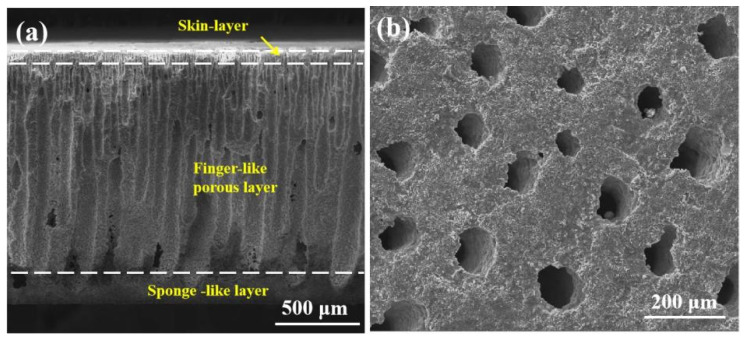
(**a**) A cross-sectional SEM image of the 3YSZ support; (**b**) the bottom view SEM image of the 3YSZ support after removing the sponge-like layer.

Figure 1a shows a cross-sectional scanning electron microscopy (SEM) image of the calcined blank 3YSZ support membrane fabricated by the phase inversion tape casting approach. The microstructure of the 3YSZ support consists of three distinct layers: a porous thin skin layer, a thick ordered finger-like hole layer in the middle, and a thin sponge layer at the bottom. The finger-like layer contains honeycomb-shaped pores with a uniform size ranging from 30 to 50 μm in diameter, as shown in Figure 1b.

**Figure 2:**
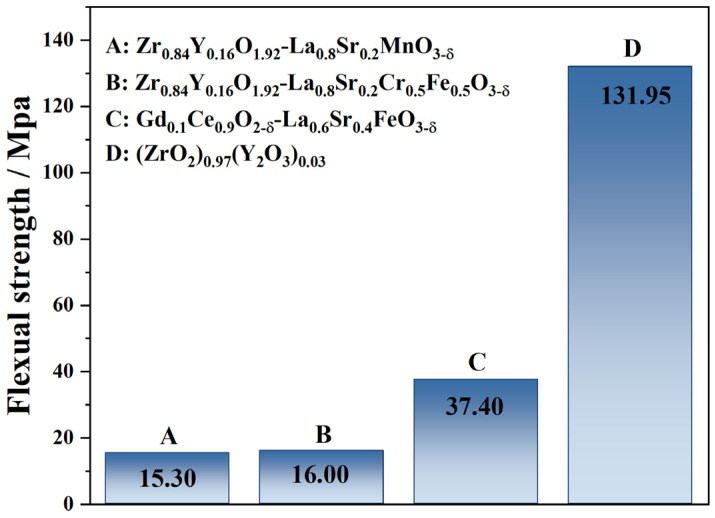
Mechanical strength comparison of various cathode supports prepared by the phase inversion method.

The 3YSZ support obtained by phase inversion tape casting was calcined at 1400 °C for 5 h and then cut into 20 × 30 mm samples for the three-point bending test. Figure 2 compares the bending–breaking strength of the 3YSZ support membrane and other conventional composite cathode membranes. It can be seen that the flexural strength of the 3YSZ support reaches high strength of 131.95 MPa compared with the Zr_0.84_Y_0.16_O_1.92_-La_0.8_Sr_0.2_MnO_3−δ_, Zr_0.84_Y_0.16_O_1.92_-La_0.8_Sr_0.2_Cr_0.5_Fe_0.5_O_3−δ_ and Gd_0.1_Ce_0.9_O_3−δ_-La_0.6_Sr_0.4_FeO_3−δ_ cathode supports [28,29], ensuring the high mechanical strength of the single cells. Previous work has also shown the enhanced mechanical strength of SOFCs using 3YSZ instead of 8YSZ, while maintaining comparable performance [30,31].

### 3.2. Electrochemical Performance

**Figure 3:**
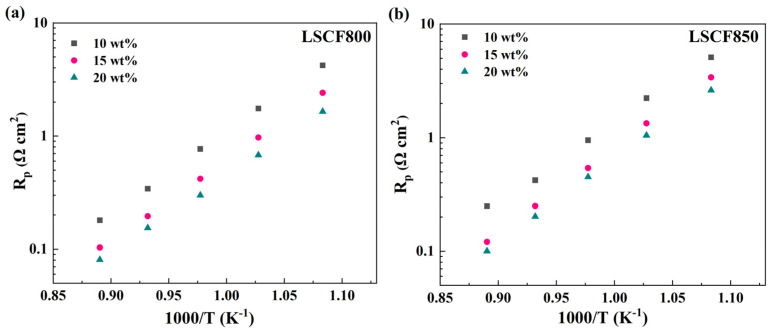
The R_p_ as a function of temperature at 650–850 °C with different LSCF catalyst loadings treated in air and O_2_ calcined at (**a**) 800 °C and (**b**) 850 °C.

Figure 3 plots the polarization resistance (R_p_) of LSCF-8YSZ/8YSZ/LSCF-8YSZ symmetrical cells measured at 650–850 °C with different catalyst loadings under different heat treatment temperatures. It can be observed that all the LSCF-infiltrated symmetrical cells (with 10 wt%, 15 wt%, and 20 wt% catalyst) after being treated at 800 °C show a much lower R_p_ compared with those heat-treated at 850 °C for 2 h. The results indicate that the optimal heat treatment temperature for the LSCF catalyst is 800 °C.

**Figure 4:**
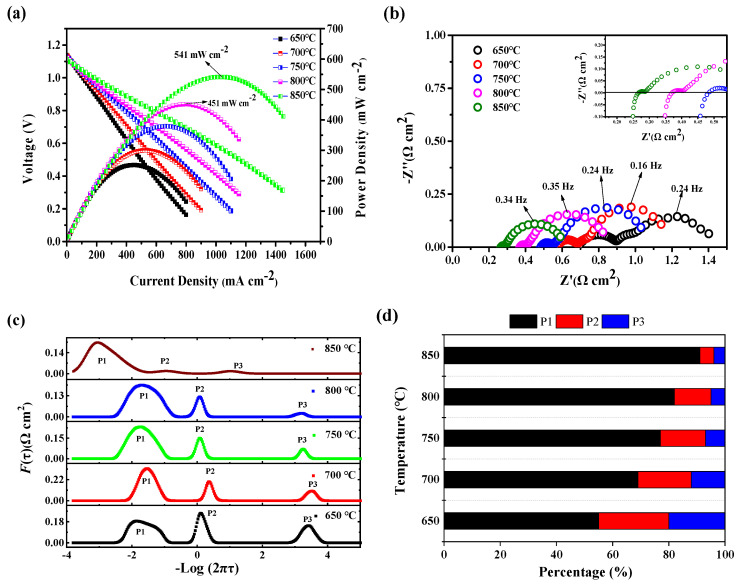
(**a**) I-V-P curves of the single cell measured at 650–850 °C; (**b**) the corresponding impedance spectra measured under OCV conditions; (**c**) DRT analysis of the EIS data measured at 650–850 °C; (**d**) the proportion of resistance corresponding to the resolution peaks.

Figure 4 shows the typical current–voltage–power (I-V-P) curves and corresponding impedance spectra of the single cell with the novel configuration of finger-like 3YSZ-LSCF, porous 8YSZ-LSCF, dense 8YSZ, and porous 8YSZ-NiO. The open circuit voltages (OCVs) of the single cell are 1.1~1.2 V within the test temperature range of 650–850 °C, which are nearly identical to the OCV values determined theoretically using the Nernst equation, indicating that the electrolyte is sufficiently dense. As seen in Figure 3a, the peak power densities of the single cell are 251, 300, 378, 451, and 540 mW cm^−2^ at 650 °C, 700 °C, 750 °C, 800 °C, and 850 °C, respectively. The performance is slightly higher than that of the LSM-YSZ cathode-supported SOFC prepared by the phase inversion tape casting method [16], demonstrating that the impregnated electrode greatly increases the triple phase boundary (TPB) length and provides more active sites for oxygen reduction reactions. Compared with the electrode prepared by the conventional screen printing process, the cell prepared by the impregnation process exhibits higher electrochemical performance [32].

The corresponding electrochemical impedance spectrum of the single cell measured under OCV conditions is shown in Figure 4b. The first intersection value between the high-frequency arc and the real axis of the spectrum is the ohmic resistance (R_o_) of the single cell. The R_o_ includes the ion transmission resistance of the electrolyte, the contact resistance of the electrode/electrolyte, and the wire resistance [33]. The silver wire was used as the current collector; therefore, the wire resistance can be essentially ignored. Meanwhile, good contact between the electrodes and electrolyte can ensure fairly low resistance. Therefore, the ohmic resistance value is mainly contributed by the electrolyte. The R_o_ values of the cell were 0.63, 0.53, 0.46, 0.35, and 0.25 Ω cm^2^ at 650 °C, 700 °C, 750 °C, 800 °C, and 850 °C, respectively, showing decreased resistance with the increasing temperature. The difference between the low-frequency intercept (the intersection of the low-frequency arc and real axis) and the high-frequency intercept (the intersection of the high-frequency arc and real axis) refers to the polarization resistance (R_p_), which includes the gas mass transmission characteristics and charge transmission resistance [34]. The charge transfer resistance is related to the electrocatalytic characteristics of the electrode materials, ion transfer velocity, TPB length, and so on [35]. The R_p_ values of the cathode-supported single cell were 0.70, 0.60, 0.57, 0.45, and 0.32 Ω cm^2^ at 650 °C, 700 °C, 750 °C, 800 °C, and 850 °C, respectively, showing decreased resistance with the increasing temperature. In addition, no obvious concentration polarization resistance was observed, suggesting better gas diffusion compared with the conventional cathode support layer prepared by the tape casting method [36].

In order to further understand the rate-limiting step of the electrochemical reactions, DRT analysis was employed to analyze the EIS of the single cell measured at 650–850 °C. Generally, the oxygen reduction reaction in the cathode side consists of oxygen adsorption, oxygen dissociation, charge transfer, and the diffusion of oxygen ions. DRT analysis can effectively separate the frequency range into individual electrochemical processes. According to the previous results, the low-frequency region of the DRT curve mainly corresponds to the gas diffusion and conversion process within the electrode of the single cell, and the medium–low-frequency region is closely related to the ORR and the oxygen ion transport in the active sites of the TPBs. Meanwhile, the high-frequency region may be associated with the charge transfer and ion transport processes at the interface between the electrolyte and electrode [34,37,38]. As shown in Figure 4c, three peaks of P1, P2, and P3 from the low frequency to high frequency are observed, respectively, which represent the kinetics of the corresponding electrode reaction at various temperatures. As shown in Figure 4c, the intensity of P1, P2, and P3 decreases with temperature, suggesting enhanced oxygen adsorption, ionic transport, charge transfer, and gas diffusion processes for the accelerated oxygen reduction reactions [18,27]. Figure 4d re-plots the proportion of simulated resistance corresponding to each peak. It can be clearly seen that the percentage of simulated resistance of P2 and P3 caused by electron–ion transmission is quite small, which means that the impregnated LSCF active electrode with sufficient electrocatalytic activity can meet the requirement for oxygen reduction reactions. On the other hand, the figure shows that P1 accounts for the highest proportion, which suggests that gas diffusion in both the anode and cathode sides is the main rate-limiting step within the test temperature range. This may be explained by the growth of nanoparticles, which causes insufficient porosity in the electrodes with the increase in temperature [39]. It has been reported that the DRT method can be employed to deconvolute the EIS data measured in various operating conditions, such variations in temperature, oxygen partial pressure, current density, elapsed time, and the amount of introduced catalyst. Based on the EIS data of the single cells and symmetrical cells measured under different parameters, it is possible to find the rate-limiting step of the electrode reactions and eventually separate the anodic and cathodic resistance [40,41]. More studies involving DRT analysis will be conducted in future work.

**Figure 5:**
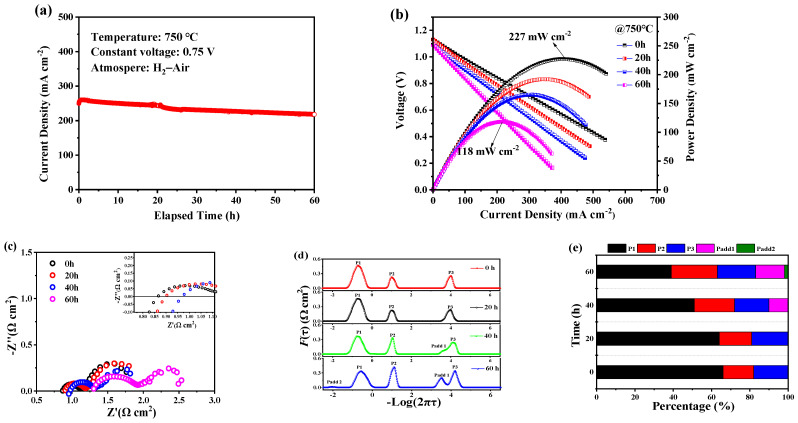
(**a**) Current density of the cell with the elapsed time under a constant voltage of 0.75 V at 750 °C; (**b**) I-V-P curves of the single cell at different measurement times; (**c**) the corresponding EIS curves at different times; (**d**) DRT analysis results of (**c**); (**e**) the proportion of resistance corresponding to the resolution peaks of (**d**).

In order to further evaluate the electrochemical properties of the prepared single cell, a durability test was conducted at a discharged voltage of 0.75 V at 750 °C in a H_2_–air atmosphere. As can be seen from Figure 5a, the current density decreases from 260 to 217 mA cm^−2^, showing a relatively fast degradation rate of 16.5% after the 60 h short-term stability test. Figure 5b compares the I-V-P curves of the single cell during the durability test at 0 h, 20 h, 40 h, and 60 h, respectively. The OCV decreases from 1.13 to 1.09 V, and the maximum power density shows almost 50% degradation from 227 to 118 mW cm^−2^ after the durability test. The corresponding impedance spectra are shown in Figure 5c. A slightly increased R_o_ and a steady R_p_ are shown within the initial 40 h. After this, a significant increase in R_o_ from 0.87 to 1.30 Ω cm^2^ and an increase in R_p_ from 0.80 to 1.3 Ω cm^2^ are observed. This increase in R_o_ and R_p_ may be ascribed to the agglomeration and coarsening of the impregnated LSCF and Ni catalysts with larger grain sizes, resulting in loose contact between the catalysts with deteriorated electronic transport and reduced TPBs for the ORR.

To further understand the degradation mechanism of the single cell, the corresponding EIS was analyzed by the DRT method. As shown in Figure 5d, no significant frequency shift is observed for all peaks in the initial 20 h. However, additional peaks of Padd1 and Padd2 can be observed after the 60 h stability test. Figure 5e re-plots the proportion of simulated resistance. There is almost no change in the peak area of P3, which is associated with the charge transfer process in the interface between the electrode and electrolyte. The peak area of P2 increases slightly, suggesting a slower three-phase interfacial redox electrochemical reaction process. An obvious area change is observed in P1, which is related to the electrode electrochemical process, showing a gradual decline in gas diffusion as well as the conversion of O_2_ to O^2-^ in TPBs. In addition, an additional peak, Padd1, appears after 40 h and shifts to a lower frequency. The distinct peak Padd1 and small peak Padd2 can be observed at 60 h. The two additional peaks indicate new rate-limiting steps, which may be due to the increased energy barrier needed to overcome the elementary reactions during long-term operation [14]. Combined with the EIS results, two factors can be considered as potential reasons for the degradation. Firstly, the agglomeration of LSCF and Ni nanoparticles during continuous operation at a high temperature of 750 °C may lead to worse gas diffusion and less TPB active sites, resulting in the increased R_p_ [42]. Secondly, the coarsening of LSCF and Ni nanoparticles in the functional layers may cause the insufficient connection of nanoparticles for electronic conduction, leading to the increased R_o_ after the durability test [43,44]. Zhou reported that the introduction of a Ce_0.8_Sm_0.2_O_3−δ_ catalyst and a low working temperature of 650 °C could effectively improve the stability of single cells, suggesting that the fabrication of composite nanocatalysts and reducing the working temperature is a promising approach to enhance the stability of single cells [45,46,47]. Future work will be focused on the optimization of the infiltrated nanocatalysts.

### 3.3. Microstructure Characterization

**Figure 6:**
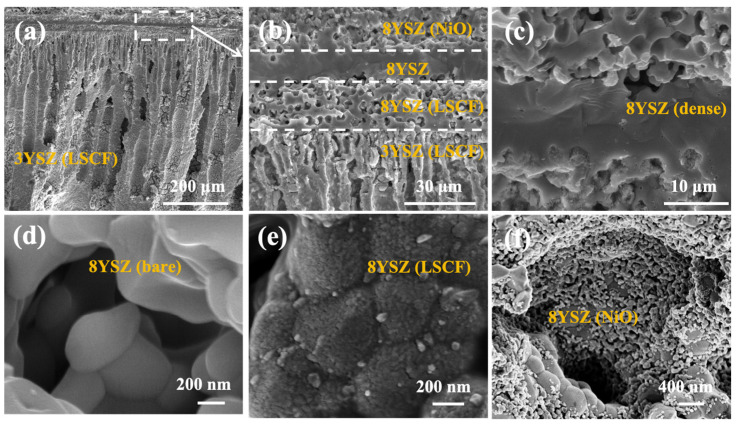
SEM images of the single cell after measurement: (**a**) overall cross-sectional view of the single cell; (**b**) partial enlarged cross-section of single cell; (**c**) electrolyte part; (**d**) locally bare porous 8YSZ skeleton without impregnation for comparison (before test); (**e**) high-magnification image of the cathode layer with impregnated LSCF nanoparticles; (**f**) high-magnification image of the anode layer with Ni nanoparticles.

Cross-sectional SEM images of an ordered porous cathode-supported SOFC consisting of 3YSZ-LSCF, 8YSZ-LSCF, dense 8YSZ, and 8YSZ-NiO after the electrochemical test are shown in Figure 6. It can be clearly seen that the supporting layer of 3YSZ-LSCF has a vertically ordered finger hole structure. During the process of the phase transformation reaction, the phase separation reaction occurred and the lean phase nuclei and rich phase nuclei of organic matter were produced due to the thermodynamic instability. After the growth and drying of the lean phase nuclei, the substrate was transformed into finger-shaped pores, as shown in Figure 6a. The whole casting body can be divided into the skin layer, finger hole layer, and sponge layer. After polishing and eliminating the sponge layer, the remaining thick finger hole layer and thin skin layer were exposed and could accelerate the gas transmission and reduce the concentration resistance [36]. Figure 6b shows a local enlarged cross-section image of the single cell. A porous–dense–porous sandwich configuration is shown in Figure 6c. The thickness of the 3YSZ supporting layer, porous 8YSZ layer, and 8YSZ electrolyte was about 700 μm, 20 μm, and 15 μm, respectively.

Previous studies have confirmed that the electrochemical performance of a single cell depends on the length of TPBs with sufficient electrochemical reaction sites and the active materials with high catalytic activity and electronic conductivity [22]. In order to better show the difference in the porous 8YSZ structure before and after impregnation, the morphology of the bare porous 8YSZ skeleton is displayed in Figure 6d. Figure 6e,f show the recorded SEM images of the impregnated LSCF-8YSZ (15 wt% LSCF) cathode functional layer and NiO-8YSZ (2 wt% NiO) anode functional layer, respectively. Both the LSCF and Ni nanoparticles are uniformly distributed, forming a continuous electron transport network, which is evenly distributed on the skeleton surface to form a cross-linked microstructure, guaranteeing sufficient conductivity and catalytic activity [48,49]. It should be noted that the electrochemical performance of the single cell may be limited by the insufficient porosity of the porous 8YSZ layer. A lower concentration of LSCF nitrate precursor solution is more conducive to infiltration, and a uniform distribution of LSCF throughout the electrode thickness in the two layers of porous 3YSZ/porous 8YSZ is essential for the fast ORR.

**Figure 7:**
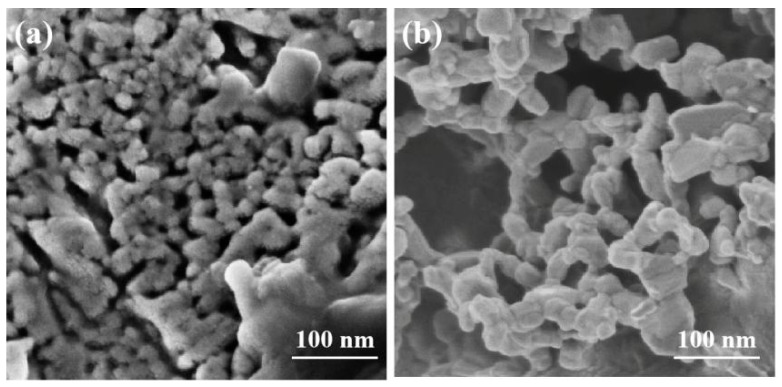
Morphology of impregnated LSCF nanoparticles: (**a**) before the durability test; (**b**) after the durability test.

Figure 7a,b present the high-magnification SEM images of the impregnated LSCF nanoparticles before and after the durability test. It can be clearly seen that the average size of the LSCF particles after the durability test is larger than that of the initially prepared ones. The degradation of the single cell is due most likely to the coarsening of the impregnated LSCF nanoparticles, which is possibly due to the lack of strong interaction between the nanoparticles and scaffolds [50,51]. In addition, the high tendency for Sr surface segregation and its migration and interaction with 8YSZ under cathodic polarization could be another reason for the degradation of the cathode [52,53]. On the other hand, Ni coarsening has been considered as the most detrimental degradation mechanism in SOFC anode electrodes, which may also lead to worse stability with both reduced TBP sites and electrical conductivity [54]. Previous work has also demonstrated the grain growth and coarsening phenomenon of Ni catalysts when the durability operation temperature is exceeded at 650 °C [46].

## 4. Conclusions

A four-layer fuel cell skeleton of finger-like porous 3YSZ, porous 8YSZ, dense 8YSZ, and porous 8YSZ with high mechanical strength was successfully prepared by the phase inversion tape casting, dip-coating, and co-sintering methods. The LSCF and Ni catalysts were fabricated by a solution impregnation method. The application of 3YSZ as a cathode support membrane offered excellent flexural strength of 131.95 Mpa. The cathode-supported SOFC achieved a peak power density of 540 mW cm^−2^ at 850 °C with an R_p_ of 0.25 Ω cm^2^. The co-sintered porous 8YSZ/dense 8YSZ/porous YSZ showed great interface bonding between the electrodes and electrolyte. DRT analysis and microstructure characterization suggested that the degradation of the single cell may result from the nanoparticles’ agglomeration and insufficient gas diffusion. The optimization of the infiltrated nanocatalysts will be the focus of future work. This work suggests a promising method to fabricate a cathode-supported cell with high strength.

## Data Availability

The data presented in this study are available on request from the corresponding author. The data are not publicly available due to ongoing researches using a part of the data.
